# A Brief Web-Based Person-Centered Care Group Training Program for the Management of Generalized Anxiety Disorder: Feasibility Randomized Controlled Trial in Spain

**DOI:** 10.2196/50060

**Published:** 2025-01-16

**Authors:** Vanesa Ramos-García, Amado Rivero-Santana, Wenceslao Peñate-Castro, Yolanda Álvarez-Pérez, Andrea Duarte-Díaz, Alezandra Torres-Castaño, María del Mar Trujillo-Martín, Ana Isabel González-González, Pedro Serrano-Aguilar, Lilisbeth Perestelo-Pérez

**Affiliations:** 1 Canary Islands Health Research Institute Foundation, Santa Cruz de Tenerife, Spain; 2 Department of Clinical Psychology, Psychobiology and Methodology, University of La Laguna (ULL), Santa Cruz de Tenerife, Spain; 3 Network for Research on Chronicity, Primary Care, and Health Promotion (RICAPPS), Tenerife, Spain; 4 The Spanish Network of Agencies for Health Technology Assessment and Services of the National Health System (RedETS), Tenerife, Spain; 5 Network for Research on Chronicity, Primary Care, and Health Promotion (RICAPPS), Madrid, Spain; 6 Área de Fomento de la Innovación e Internacionalización de la Investigación Sanitaria, Subdirección General de Investigación Sanitaria y Documentación, Dirección General Investigación y Docencia, Consejería de Sanidad, Madrid, Spain; 7 Evaluation Unit (SESCS), Canary Islands Health Service (SCS), Santa Cruz de Tenerife, Spain

**Keywords:** person-centered care, primary care, shared decision-making, anxiety disorder, training program, SDM

## Abstract

**Background:**

Shared decision-making (SDM) is a crucial aspect of patient-centered care. While several SDM training programs for health care professionals have been developed, evaluation of their effectiveness is scarce, especially in mental health disorders such as generalized anxiety disorder.

**Objective:**

This study aims to assess the feasibility and impact of a brief training program on the attitudes toward SDM among primary care professionals who attend to patients with generalized anxiety disorder.

**Methods:**

A feasibility randomized controlled trial was conducted. Health care professionals recruited in primary care centers were randomized to an intervention group (training program) or a control group (waiting list). The intervention consisted of 2 web-based sessions applied by 2 psychologists (VR and YA), based on the integrated elements of the patient-centered care model and including group dynamics and video viewing. The outcome variable was the Leeds Attitudes Towards Concordance scale, second version (LATCon II), assessed at baseline and after the second session (3 months). After the randomized controlled trial phase, the control group also received the intervention and was assessed again.

**Results:**

Among 28 randomized participants, 5 withdrew before the baseline assessment. The intervention significantly increased their scores compared with the control group in the total scale (*b*=0.57*; P*=.018) and 2 subscales: communication or empathy (*b*=0.74; *P*=.036) and shared control (ie, patient participation in decisions: *b*=0.68; *P*=.040). The control group also showed significant pre-post changes after receiving the intervention.

**Conclusions:**

For a future effectiveness trial, it is necessary to improve the recruitment and retention strategies. The program produced a significant improvement in participants’ attitude toward the SDM model, but due to this study’s limitations, mainly the small sample size, more research is warranted.

## Introduction

About 264 million people in the world are affected by anxiety disorders, according to the latest estimates of the World Health Organization [1]. In Spain, around 2 million people (4.1% of the population) suffer from anxiety disorders [1]. In primary care (PC) settings, the generalized anxiety disorder (GAD) is one of the most prevalent anxiety disorders [2]. GAD is characterized by a continuous state of worry and alertness most of the time [3] and sometimes, its high comorbidity with other psychiatric and somatic disorders makes diagnosis difficult [4]. GAD has a tendency to chronicity, due to its specific characteristics, leading to the person being worried and alert most of the time [3]. Information on the causes of the disorder and the available treatments is an unmet need in this population, given that some patients with GAD are willing to have an active or collaborative role in their health care [5].

Person-centered care (PCC) is considered the gold standard for medical care in health care settings because it humanizes the person and places him or her at the center of clinical decision-making [6]. The PCC model consists of several components, one of which is shared decision-making (SDM), whose goal is to create a collaborative dialogue between patients and health care professionals, in which patients’ values, preferences, and concerns about the different available treatment options are taken into account and incorporated into the decision-making process [7-9].

Patient decision aids are tools designed to facilitate SDM. Its use can help patients participate in the clinical decisions, improving the decision-making process and promoting informed decisions that are concordant with patients’ values and preferences [10]. On the part of professionals, it is important to develop communication skills and empathy to help patients participate in the decisions [11-13]. Research has shown that interventions and training programs aimed to promote the PCC model may improve professionals’ knowledge and the ability to communicate with patients [12,14] as well as patients’ satisfaction [15]. However, there are some barriers to apply the PCC model related to time constraints, clinical uncertainty, poor expectations, patients’ characteristics (eg, age, comorbidity, and attitude), lack of continuity of care, or knowledge about SDM [16-19]. Despite some SDM training programs have been developed for health care professionals, very few of them have been evaluated [20-22]. Therefore, despite the growing acceptance of interventions to implement SDM in health care settings, several gaps remain in the demand, perception, and clinical application of the PCC model [23,24]. In mental health care, and specifically in GAD, interventions to promote the SDM process are still very limited [25,26]. A recent qualitative study with patients with GAD concluded that there is scarce orientation to elicit patients’ preferences and values throughout the process of care [27], emphasizing the need of interventional studies aimed at promoting SDM in the clinical encounter.

The aim of this study is to evaluate the feasibility and effect of a brief training program on the attitudes toward SDM for professionals in PC who attend patients with GAD.

## Methods

### Design

A feasibility randomized controlled trial (RCT) was conducted, in which participants were allocated to a PCC training program or a control group (waiting list). It was carried out in 13 PC centers in Tenerife (Canary Islands, Spain), from January 2021 to February 2022.

### Ethical Considerations

The study was approved by the ethics committee of the Hospital Universitario Nuestra Señora de La Candelaria (reference: CHUNSC_2019_58). The study was not registered because participants were health professionals and not patients, the intervention was educational, and the only outcome measured was attitudinal. Participants who agreed to participate signed a web-based informed consent form.

### Participants

Participants were health care professionals working in PC centers (ie, physicians and nurses) or community mental health units (ie, psychiatrists, psychologists, and nurses) for at least 1 year before the start of the study, who attend patients with GAD in the Canary Islands, Spain. There were no exclusion criteria.

### Procedure, Randomization, and Allocation Concealment

The directors of the health centers were contacted and informed about the study. They were asked to invite the professionals from their centers to participate. The invitation included an infographic, graphically describing the study and a link to a web platform, where health professionals could register their willingness to participate and contact information. Then, they were contacted by telephone to provide a full explanation of the study. Those who agreed to participate signed a web-based informed consent form (reference: CHUNSC_2019_58). Participants were randomly assigned to either the intervention or control group (waiting list), using a computer-generated random number table. The randomization process was conducted by an independent researcher who was not involved in the recruitment or assignment of participants. In addition, the researcher who recruited the professionals was blinded to the group assignments in order to maintain allocation concealment. Due to the nature of the intervention, the study participants could not be blinded.

### Intervention

Intervention group participants received 2 training sessions via Zoom (version 5.15.7. [21404]) based on the integrated elements of the PCC model [28]. The training was originally intended to be applied in person, in a group format, but this was not possible due to the COVID-19 pandemic, so it was finally applied on the web. Sessions were conducted by 2 researchers (VR and YA [psychologists]). The first session lasted approximately 2 hours and was focused on presenting the principal elements of intervention: (1) introduction, which included a description of common clinical relationship models (first 20 minutes); (2) basic characteristics of the basic PCC model, through group dynamics and video viewing of a role-play in the clinical practice with a patient with GAD; this included a description of the Feelings, Ideas, Function, and Expectations model [29] (60 minutes), which was developed at the University of Western Ontario and explores the patient’s emotions, his or her ideas on what caused the problem, the effects of the illness on his or her functioning and relationships, and his or her expectations for the future and from medical care [29,30]; and (3) presentation of the Three-Talk Model for SDM, a multistage consultation process developed by Elwyn et al [31] (30 minutes). The Three-Talk Model for SMD is a theoretical approach that describes collaborative deliberation. It outlines 3 broad steps that form the core elements of SDM [31]. The last 10 minutes of the session were aimed at the resolution of doubts. The detailed contents of this first SDM training session are shown in Table 1.

**Table 1.** Content of first shared decision-making (SDM) training session.

Module and content	Form of communication	Learning objectives
Introduction		
Clinical relationship models	Lecture Video examples Interactive live Feedback with group dynamic	Be able to know the characteristics of the paternalistic, informative or contractual, interpretive or personalized, and deliberative or friendly models
Characteristics of a basic PCC^a^ model		
Explore the disease	Lecture	Acquire skills in active listening and directed anamnesis in the use of SDM
Know the patient’s perspective (beliefs, fears, expectations, repercussions, etc)	Lecture Video examples Interactive live Feedback with group dynamic	Acquire skills in how to prepare the ground and how to explore the personal experience of the disease in terms of SDM Be able to use the FIFE^b^ model to improve the quality of communication in terms of SDM
Know the person (“moving from patient to person”)	Lecture Video examples	Acquire skill about how exploring the personal and social context of the disease in terms of SDM
Involve the patients in their disease	Lecture Video examples Interactive live Feedback with group dynamic	Acquire information skills to reach agreements on problem solving, to seek shared solutions, and to involve the patient in the use of SDM
Three-Talk Model for SDM		
Team dialogue	Lecture	Acquire skills to establish a team dialogue based on the needs for change on beliefs and preferences
Dialogue on options	Lecture	Acquire skills to discuss the treatment options that exist for the disease
Dialogue on the decision	Lecture	Acquire skills to help the patient decide on which option to choose

a PCC: person-centered care.

b FIFE: Feelings, Ideas, Function and Expectations.

The second session was carried out 3 months later (review session), with an approximate duration of 1 hour. The structure of the session included (1) the review of the main contents of the first training module, together with comments on participants’ potential and sharing their experiences applying the SDM model since then (30 minutes), and (2) the discussion on the main barriers and facilitators for patients and professionals in applying the SDM process in the clinical practice (30 minutes). Detailed content of this session is present in Table 2.

Control group participants did not receive any intervention. They were informed that they could access the training program after the feasibility RCT was completed. Participants completed the baseline and 3-month (postintervention) assessments. Subsequently, participants in the control group received the intervention and were reevaluated 3 months later (second postintervention measure).

**Table 2.** Content of second shared decision-making (SDM) training session.

Unit and content	Form of communication	Learning objectives
(1) Introduction and (2) characteristics of a basic PCC^a^ model		Review the characteristics of the paternalistic model,: informative or contractual, interpretive or personalized, and the deliberative or friendly models Review tasks in active listening and directed anamnesis in the use of SDM: how to prepare the ground and how to explore the personal experience of the disease; how to explore the personal and social context of the disease; and how to reach agreements on problem solving, to seek shared solutions, to involve the patient-shared solutions, and to involve the patient in the use of SDM
Clinical relationship models Explore the disease; know the patient’s perspective (beliefs, fears, expectations, repercussions, etc); know the person (“moving from patient to person”); and involve the patients in their disease	Lecture	
(3) Characteristics of the Three-Talk Modelfor SDM		Be able to apply the principal components of the Three-Talk Model for SDM Have knowledge about how to apply this model in clinical practice to support SDM
Fifteen characteristics total of a Three-Talk Model for SDM are described: *First step*: Take a step back, present the possibility of choice, justify the choice, personalizing preference, uncertainty, check the reaction, and postpone closure *Second step*: Check knowledge, list of options, provide decision support to the patient, and summaries *Third step*: Focus on preferences, elicit a preference, lead toward a decision, and offer review	Lecture	
(4) Barriers and enablers to apply Three-Talk Model for SDM		Invite to participate by presenting the experience from a professional point of view in clinical practice Openly share and discuss observations of the professional communication Offer, explicitly and without judging, feedback on implementation
Identification of barriers from a professional point of view that can condition the application of the 3-step model for SDM	Identification of professionals' own barriers to communication with their patients	
Identification of barriers from a patient’s point of view that can condition the application of the 3-step model for SDM	Identification of patients' own barriers to communication with their care team	
Identification of facilitators who may exist to carry out the 3-step model for SDM	Identify the individual facilitators in communication to implement a SDM model	

a PCC: person-centered care.

### Measures

The outcome measure was the professionals’ attitude toward PCC. It was assessed with the Leeds Attitudes Towards Concordance scale, second version (LATCon II) [32]. This self-report instrument includes 20 items with a 4-point Likert format from strongly disagree (0) to strongly agree (3). Although the original instrument includes 5 subscales, we used the 3 components identified by means of principal component analysis in the Spanish validation [33], carried out with psychiatrists and psychiatry residents. These subscales were labeled “communication/empathy” (CE, 12 items about the importance of a good communication and the consideration of patient’s feelings and beliefs), “shared control” (SC, 4 items reflecting a positive attitude toward equality and SDM), and “eventual paternalistic style” (EPS, 4 items stating that sometimes a paternalistic style is necessary; these items are reverse-coded, and therefore higher scores indicate lower agreement with EPS) [33]. Scores on the total scale and the subscales are divided by the corresponding number of items, thus ranging 0‐3. The LATCon II has shown good internal consistency in previous studies [33-35].

The following sociodemographic and professional variables were measured at baseline: age, gender, specialty (medicine or nursing), years of professional experience and work in the health care center, level of perceived workload (low, medium, and high), and previous training on PCC or SDM.

### Statistical Analysis

We calculated that a mixed model with 2 repeated measures per participant (cluster) requires 38 subjects (19 in each group) in order to detect a significant moderate-to-strong between-group effect (standardized mean difference of 0.80), assuming type I and II errors of 0.05 and 0.20, respectively, and an intraclass correlation of 0.50 [35].

Descriptive statistics were calculated for continuous and categorical variables (means, SDs, and percentages). Cronbach α was calculated for the LATConII scale and its 3 subscales, as well as the correlations between the subscales (Spearman ρ). The effect of the intervention was analyzed with mixed lineal models, including fixed effects for time (pre, post), group (intervention, control) and its interaction, and the participant as a random effect (assuming an unstructured covariance matrix). Successive models were carried out adjusting for 1 covariate at a time (ie, sociodemographic and professional variables). Unstandardized β values and effect sizes (Hedges *g*) are reported.

Changes from baseline to postintervention were evaluated analyzing the effect of time in a mixed model separately for each group. The same test was used to analyze the change in the control group after receiving the intervention upon completion of the RCT. Analyses were performed with SPSS (version 25; IBM Corp) and STATA (version 17; StataCorp LLC).

## Results

**Figure 1.**
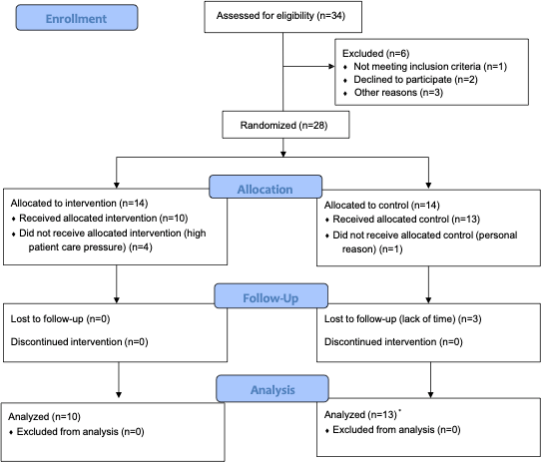
CONSORT flow diagram. *The analysis includes 3 control participants lost at postintervention.

Thirty-four health professionals were interested in participating and were contacted by phone. After being informed in detail, 6 declined participation and 28 accepted, signing informed consent and being randomly allocated to the intervention or control group (14 each). However, 5 of them (4 in the intervention group) withdrew from the study before completing the baseline assessment (Figure 1). Table 3 shows the characteristics of the 23 participants. There were 18 women (18/23, 78.3%) and the mean age was 48.3 (range: 26‐64) years. They had an average of 22.3 years of professional experience, and 52% (12/23) considered having a high caseload. Only 5 (21.7%) had had previous training in PCC.

**Table 3.** Characteristics of participants.

	Intervention (n=10)	Control (n=13)	Total (N=23)
Female, n (%)	10 (100)	8 (61.54)	18 (78.26)
Age, mean (SD)	45.60 (10.82)	50.38 (9.77)	48.30 (10.24)
Specialty, n (%)			
Nursing	3 (30)	2 (15.38)	5 (21.74)
Medicine	7 (70)	11 (84.62)	18 (78.26)
Years of professional experience, mean (SD)	19.70 (9.44)	24.33 (9.42)	22.32 (9.51)
Years working in the center, mean (SD)	7.05 (7.15)	6.40 (7.02)	6.68 (6.92)
Previous training in PCC^a^, n (%)	1 (10)	4 (30.77)	5 (21.74)
Self-perceived care load, n (%)			
Low-medium	6 (60)	5 (38.46)	11 (47.83)
High	4 (40)	8 (61.54)	12 (52.17)

a PCC: person-centered care.

At baseline, internal consistency (Cronbach α) was 0.94 for the total LATConII scale, and 0.97 (CE), 0.88 (SC), and 0.25 (EPS) for the subscales. The total mean score was 2.08 (SD 0.60), and the mean scores were 2.29 (SD 0.78), 1.77 (SD 0.85), and 1.78 (SD 0.41) for the subscales CE, SC, and EPS, respectively (Table 3). CE and SC were significantly correlated (ρ=0.49; *P*=.01), whereas EPS was not significantly associated with CE (ρ=0.11; *P*=.62) or SC (ρ=0.29; *P*=.180) (Table 4).

**Table 4.** Effect of the intervention.

Time^a^	Intervention(n=10), mean (SD)	Control(n=10), mean (SD)	Time × group interaction,*b* (*P)*^b^	Between-group effect size, Hedges *g* (95% CI)
LATCon II^c^ total (range: 0‐3)			0.57 (.018)	0.92 (0.13 to 1.71)
Pre	1.87 (0.76)	2.25 (0.40)^d^		
Post	2.27 (0.51)^e^	2.08 (0.61)		
Post2	—^f^	2.60 (0.24)^g^		
Communication/empathy (range: 0‐3)			0.74 (.036)	0.86 (0.06 to 1.65)
Pre	1.98 (1.02)	2.52 (0.46)^d^		
Post	2.57 (0.70)^e^	2.34 (0.86)		
Post2	—	2.84 (0.20)^e^		
Shared control (range: 0‐3)			0.68 (.040)	0.76 (0.01 to 1.52)
Pre	1.55 (1.06)	1.94 (0.63)^d^		
Post	1.80 (0.44)	1.52 (0.70)^e^		
Post2	—	2.28 (0.43)^h^		
Eventual paternalistic style (range: 0‐3)			−0.04 (.856)	0.08 (−0.93 to 0.93)
Pre	1.83 (0.44)	1.75 (0.41)^d^		
Post	1.83 (0.57)	1.83 (0.54)		
Post2	—	2.18 (0.64)^e^		

a Pre-post: randomized controlled trial (intervention vs waiting list); post2: intervention period for the control group, after the randomized controlled trial.

b Unstandardized β coefficients (*P* value) from mixed lineal models analyzing the randomized controlled trial (pre-post), including the participant as a random effect (the analysis includes 3 control participants lost at postintervention).

c LATCon II: Leeds Attitudes Towards Concordance scale, second version.

d n=13.

e *P<*.05.

f Not applicable.

g *P*<.001, compared with the previous assessment (effect of time in mixed models separately by group).

h *P<*.01.

Three control participants were lost at postintervention (3 months), but their baseline scores were included in the mixed models on an intention-to-treat basis (postintervention scores were not imputed). The time × group interaction was statistically significant for the total scale, showing a differential increment in scores favoring the intervention (*b*=0.57; *P*=.01) (Table 4). The same occurred with the subscales CE (*b*=0.74; *P*=.036) and SC (*b*=0.68; *P*=.04). The inclusion of potential confounders in the model did not change the results (see Table S1 in Multimedia Appendix 1 for the total scale). The intervention group significantly increased their scores compared with baseline in the total scale (*b*=0.4; *P*=.033) and CE (*b*=0.58; *P*=.030), whereas the control group significantly decreased in SC (*b*=−0.43; *P*=.037) (Table 4).

After the trial was completed, the control group received the intervention and showed significant increments in the total score (*b*=0.52; *P*<.001) and the 3 subscales: CE (*b*=0.50; *P*=.020), SC (*b*=0.75; *P*=.002), and EPS (*b*=0.35; *P*=.02) (Table 4).

## Discussion

### Principal Findings

This study aimed to evaluate the feasibility and effect of a brief web-based training program on the attitudes toward SDM and PCC of PC professionals who treat patients with GAD. The program was initially intended to be conducted in person at the professionals’ centers, but due to the pandemic context, it was shifted to a web-based format. The 2 sessions went smoothly and the professionals actively participated, asking questions and describing their experiences related to SDM. Previous studies evaluating learning programs for health professionals or university students have not shown relevant differences between web-based and in-person formats [36-39], although in some cases better results have been observed with the face-to-face intervention [40]. Given the brevity of our program, we do not expect that there will be relevant differences between both formats.

The recruitment and retention rate were low, only 33 eligible professionals showed interest in the study (2.5 per center) during the 5-month recruitment period, and 5 declined participation when they were fully informed about the study. It is possible that direct contact with professionals, instead of the general call that was made through center directors, would have improved the recruitment rate to some extent. Among the 28 randomized participants, 5 more did not start the trial and 3 did not complete the study. The high workload, a common situation in the Spanish public health system even in a nonpandemic context, was the main reported cause of these withdrawals. On the other hand, the group format enriches the training process by enabling the interaction of professionals, but it also represents a difficulty when coordinating their schedules and availability. In summary, the participation and retention rates were not satisfactory, and for future trials it is necessary to develop more structured and intensive strategies. Theoretical frameworks as proposed by Solberg [41] that identified 7 factors that influence the recruitment of health care professionals (ie, relationships, reputation, requirements, rewards, reciprocity, resolution, and respect) could help to this aim.

Regarding effectiveness, the results showed significant moderate-to-strong effects (although with very wide confidence intervals) on the total scale and the CE and SC subscales. The pre-post change in the intervention group was greater on the former, and the similar between-group effect size was due in part to a significant decrease in SC in the control group. The EPS dimension was not affected by the intervention, but this result is unclear given the low internal consistency of this subscale (future studies should confirm the factorial structure of the instrument). After the RCT was completed, the control group received the intervention and showed significant before-after improvements of similar magnitude in the 3 dimensions. Due to the wide confidence intervals, the results should be interpreted with caution and verified in studies with greater statistical power.

Baseline scores indicated a positive attitude (values above the midpoint of the scale) for the total scale and the 3 subscales, although scores on CE and SC suggest that, comparatively, participants seemed more favorable to empathetically communicate with their patients than sharing decisions with them. This result has also been observed in several studies that applied the Patient‐Practitioner Orientation Scale [42], the most frequently used instrument to assess health professionals’ attitudes toward PCC, showing higher scores on the *caring* subscale of the questionnaire (ie, empathy, warmth, and treating patients as whole persons) than on the *sharing* one (ie, sharing information, decisions, and power) [43-47].

Other studies also have shown significant benefits of different training programs on professionals’ and medical students’ attitudes toward SDM and PCC and their intention to apply it in the future, showing high levels of satisfaction with the program [48-52]. A positive attitude toward the PCC model is an obvious requisite for the professionals’ learning and demonstration of behaviors aimed at promoting SDM in consultation. Validation studies with the Patient‐Practitioner Orientation Scale showed that more favorable attitudes were significantly associated with more patient‐centered behaviors in consultations [53], and that concordance of patients and physicians’ attitudes was associated with greater patient’s satisfaction [53-55], trust, and endorsement of physicians [53], as well as fewer referrals to specialized care [56]. Nonetheless, for the implementation of SDM it is necessary to have not only a positive attitude toward PCC but also the appropriate knowledge and communication skills required by this model, for which training programs have been developed. However, the effect of interventions targeting health professionals on the actual promotion of SDM in consultation remains uncertain. The last update of a Cochrane systematic review reported a significant effect of these interventions (eg, educational meetings and materials, outreach visits, and reminders), compared with usual care when SDM in consultation was assessed by external observers, but not by patients, even when the intervention is directed to both patients and professionals [11]. Observational studies have also shown a lack of association between patients’ and external observers’ perception of SDM [57-59], but the causes of this discrepancy have not been investigated. Furthermore, the evidence about the effects of SDM interventions targeting health professionals on patients’ cognitive, affective, behavioral, and health outcomes is also scarce [10].

Although the PCC and SDM models are a paradigm to be applied to every patient regardless of his or health problems, patients with GAD could present specific psychological characteristics that might affect the decision-making process. In experimental settings involving stimulus reinforcement, these patients have shown greater intolerance to uncertainty and impaired decision-making [55,57-59]. Nonetheless, this does not translate into a preference for a passive role in decision-making, since a recent study showed that more than 80% research participants desired to play an active or collaborative role when making decisions about treatment, although one-third of them perceived more involvement than they preferred [60]. Therefore, professionals should adapt the SDM process to the patients’ preference for involvement and manage the unavoidable uncertainty about the potential adverse effects of treatment and the likelihood and intensity of symptoms’ improvement.

### Limitations

The study has important limitations. First, feasibility of in-person group sessions could not be evaluated due to the emergence of the COVID-19 pandemic, but that allowed us to check the web-based application of the program, which was delivered without problems. However, the recruitment and retention rates were low. The recruited sample was small and there were some relevant differences in baseline variables, including the scores on the LATCon, and therefore a high risk of selection bias is present. The intervention group was 5 years younger and less experienced, included more nurses and less participants with prior experience on SDM training, and showed a less favorable attitude toward SDM. These characteristics suggest a greater margin for potential benefit in this group. Although the inclusion of these covariates in the model did not change the results, this analysis is strongly underpowered. Nonetheless, given the strong effects sizes obtained and the similar ones showed by the control group after receiving the training, it is reasonable to think that the intervention could produce a real improvement in attitudes, although effects sizes are probably inflated due to the mentioned confounders. The small sample size and the fact that participants were voluntary also challenges the external validity of the results, since it is probable that they were more motivated or favorable to the SDM model.

On the other side, this was a pilot study and we did not assess other professionals’ outcomes (eg, knowledge of SDM, satisfaction with the program, and intention to apply SDM in the future), whether the observed effect is maintained over time or its influence on professionals’ behavior in consultation as well as on patients’ outcomes, which is the ultimate aim of these interventions. An RCT with an adequate sample size is warranted to confirm the results on professionals’ attitude and to investigate the mentioned issues.

## Supplementary material

10.2196/50060 Multimedia Appendix 1 Results of the models including covariates on the LATCon total score.

10.2196/50060 Checklist 1. CONSORT-EHEALTH checklist (V 1.6.1).

## Abbreviations

CE: communication/empathy
EPS: eventual paternalistic style
GAD: generalized anxiety disorder
LATConII: Leeds Attitudes Towards Concordance scale, second version
PC: primary care
PCC: person-centered care
RCT: randomized controlled trial
SC: shared control
SDM: shared decision-making
