# Supplementary material for: A Brief Web-Based Person-Centered Care Group Training Program for the Management of Generalized Anxiety Disorder: Feasibility Randomized Controlled Trial in Spain
Source: JMIR Med Educ. 2025 Jan 16;11:e50060. doi: 10.2196/50060 (PMC11756839; doi:10.2196/50060)
Supplement: Multimedia Appendix 1 [file mededu-v11-e50060-s001.pdf]

Table S1. Results of the models including covariates on the LATCon total score.

| | **Effect of the covariable**<br>**B (95%CI)**<br>**p-value** | **Time x Group**<br>**B (95%CI)**<br>**p-value** |
|---|---|---|
| Age | <0.01 (-0.02, 0.02)<br>*P*=.997 | 0.58 (0.09, 1.06)<br>*P*=.020 |
| Gender | 0.60 (0.03, 1.17)<br>*P*=.037 | 0.58 (0.09, 1.06)<br>*P*=.020 |
| Nurse/physician | 0.17 (-0.39, 0.73)<br>*P*=.551 | 0.58 (0.09, 1.06)<br>*P*=.020 |
| Years of experience | 0.13 (-0.30, 0.57)<br>*P*=.549 | 0.58 (0.09, 1.06)<br>*P*=.020 |
| Previous SDM training | 0.29 (-0.23, 0.81)<br>*P*=.273 | 0.58 (0.09, 1.06)<br>*P*=.020 |
| Care load | -0.03 (-0.49, 0.43)<br>*P*=.889 | 0.58 (0.09, 1.06)<br>*P*=.020 |

Note: data show the unstandardized Beta (95%CI) from models including the covariate and the time x group interaction (one model for each covariate).
CI: confidence interval.
